# Siderophore-Producing Bacteria from a Sand Dune Ecosystem and the Effect of Sodium Benzoate on Siderophore Production by a Potential Isolate

**DOI:** 10.1100/2012/857249

**Published:** 2012-05-02

**Authors:** Teja Gaonkar, Pramoda Kumar Nayak, Sandeep Garg, Saroj Bhosle

**Affiliations:** Department of Microbiology, Goa University, Taleigao Plateau, Goa 403 206, India

## Abstract

Bioremediation in natural ecosystems is dependent upon the availability of micronutrients and cofactors, of which iron is one of the essential elements. Under aerobic and alkaline conditions, iron oxidizes to Fe^+3^ creating iron deficiency. To acquire this essential growth-limiting nutrient, bacteria produce low-molecular-weight, high-affinity iron chelators termed siderophores. In this study, siderophore-producing bacteria from rhizosphere and nonrhizosphere areas of coastal sand dunes were isolated using a culture-dependent approach and were assigned to 8 different genera with the predominance of *Bacillus* sp. Studies on the ability of these isolates to grow on sodium benzoate revealed that a pigmented bacterial culture TMR2.13 identified as *Pseudomonas aeruginosa* showed growth on mineral salts medium (MSM) with 2% of sodium benzoate and produced a yellowish fluorescent siderophore identified as pyoverdine. This was inhibited above 54 **μ**M of added iron in MSM with glucose without affecting growth, while, in presence of sodium benzoate, siderophore was produced even up to the presence of 108 **μ**M of added iron. Increase in the requirement of iron for metabolism of aromatic compounds in ecosystems where the nutrient deficiencies occur naturally would be one of the regulating factors for the bioremediation process.

## 1. Introduction

Sand dune ecosystems are described as mounds of sand with vegetation, found along the coastal areas [1]. Dunes are characterized by low nutrients, drought, high salinity, and sand erosion. Plants growing in sand dune habitat help in stabilization of the same, and are controlled by the interaction between, biotic and physicochemical components of sand matrix [2]. Low iron content is a peculiar feature of rhizosphere of such plants. Iron-limiting conditions influence the efficiency of microbial activity and alter utilization of various compounds by microorganism. The most apparent example is the microbial degradation of aromatic compounds as oxygenases involved in hydrocarbon degradation contain iron as cofactor, thus imposing specific iron requirement on cells [3]. Under such conditions, microorganisms scavenge extracellular iron by secreting iron-binding compounds called siderophores.

Coastal sand dune plants harbor diverse community of microbes associated with their rhizosphere and roots [4, 5]. The interactions between plants and bacteria help plants to settle in ecosystem restoration process [6, 7]. Plant microbe symbioses have been exploited in programs of sand dune restoration. Arbuscular mycorrhizal fungi important to some sand dune plants have been used in restoration projects of coastal sand ecosystems [8]. Despite the role played by bacterial diversity in sand dune plant communities, very few reports are available on the distribution and abundance of rhizosphere-associated bacteria. Park et al. [5] have isolated and characterized bacteria associated with sand dune plant spp. from Korea and further characterized their plant growth-promoting potential [9]. Arun and Sridhar [2] have studied symbiotic performance of fast growing rhizobia isolated from costal sand dunes of Karnataka, India. Microorganisms associated with plants augment their growth by increasing root development, nitrate uptake, solubilizing phosphorous, or by controlling soil-borne pathogens by competing for limiting nutrients [10]. Siderophore producers indirectly promote plant growth by competing with plant pathogens for iron [11]. A number of plants possess heterologous iron uptake mechanism for acquisition of iron through iron-bacterial siderophore complex [12].

In our earlier studies, we have reported plant growth promotion by bacterial isolates from rhizosphere of *Ipomea pes-caprae* and *Spinifex littoreus *[13]. The present study was undertaken to investigate the distribution of siderophore-producing bacteria in rhizosphere and nonrhizhosphere regions of coastal sand dunes of Miramar, India. Furthermore, we also screened these isolates to determine their ability to degrade sodium benzoate.

## 2. Materials and Methods

### 2.1. Sample Collection and Determination of Viable Counts

Sand samples were collected from coastal sand dunes of Miramar, Goa located on the west coast of India. Rhizosphere samples were collected from rhizosphere of sand dune creeper *Ipomoea pes-caprae* and nonrhizosphere samples from areas devoid of sand dune vegetation. Sand dune samples were serially diluted in physiological saline and plated on two different media, nutrient agar (NA) and Tryptone yeast extract glucose agar (TYG). Plates were incubated at room temperature and examined after 24 and 48 hrs for bacterial colony-forming units (cfu g^−1^). Viable counts were recorded, and predominant isolates were purified and maintained on slants at 4°C.

### 2.2. Determination of the Iron Content of the Samples

The digestion of the sand samples for determining iron content was carried out as described by I. Jarvis and K. E. Jarvis [14]. Briefly, 0.2 g of sand sample was digested with 10 mL of the mixture of hydrofluoric acid : nitric acid, and perchloric acid (7 : 3 : 1) and dried on hot plate at 150°C. After drying, 5 mL of the above mixture was added and dried on hot plate for 1 hour. 2 mL of concentrated HCl was added and dried completely. Residue was dissolved in 10 mL of 1 : 1 HNO_3_, and the contents were diluted to 25 mL with milli Q water. The concentration of Fe was determined by AAS (Shimadzu 6300).

### 2.3. Screening for Siderophore Producers

Siderophore production was determined using CAS assay [15]. The medium NA/TYG deferred by adding 8-hydroqxyquinoline dissolved in chloroform to ensure complete removal of Fe [16]. The isolates were spot inoculated, and plates were incubated till 72 hours, and the isolates forming yellow zone were selected for further studies. 

### 2.4. Selection and Identification of the Isolates

The cultural, morphological, and biochemical characteristics of the isolates producing siderophores were determined, and the isolates were identified based on Bergey's Manual of Systemic Bacteriology [17, 18]. In addition to biochemical analyses, nine isolates were subjected to partial sequencing of 16S rRNA gene. The genomic DNA was extracted as described by Sambrook et al. [19]. 16S rRNA gene was amplified using standard universal forward primer (S-D-Bact-0011-a-5-17: 5′-GTTTGATCCTGGCTCAG-3′) and standard universal backward primer (S-∗-Univ-1392-b-A-15: 5′-ACGGGCGGTGTGTNC-3′). The derived 16S rRNA gene sequence was compared with sequences in the GenBank database using the BLAST search program [20] and aligned by using the multiple alignment Clustal X program [21].

### 2.5. Screening of the Siderophore Producers for Sodium Benzoate Degradation

Selected isolates were streaked on mineral salts medium (MSM) [22] supplemented with various concentrations of sodium benzoate (0.1%, 0.2%, 0.5%, 0.75%, 1%, 1.5%, and 2% concentrations). The plates were incubated at 28°C up to seven days.

### 2.6. Spectrophotometric and Spectrofluorimetric Characteristics of Siderophore Produced by the Isolate TMR2.13

TMR2.13 was inoculated in MSM with 0.2% of glucose, and the flask was incubated at 28°C on shaker at 100 rpm for 24 hours. The culture broth was centrifuged, and the supernatant was scanned for peaks in UV-Vis range using spectrophotometer (Shimadzu UV-2450). Fluorimetric analysis was carried out with RF-5301 PC Shimadzu spectrofluorometer at excitation and emission wavelengths of 400 and 467 nm, respectively. Fluorescence quenching was studied by adding 10 *μ*L of Fe^+2^/Fe^+3^ solutions to 3 mL of crude culture supernatant to achieve a final concentration of 3.3 *μ*M [23].

### 2.7. Evaluation of Siderophore Production in Presence of Sodium Benzoate in MSM

Flasks containing MSM with 0.2% of glucose or sodium benzoate as the sole carbon source, supplemented with iron, in increasing concentration (0, 13.5, 27, 54, 108, 216, and 432 *μ*M) were inoculated with 5% of exponential cells grown in the respective medium. All the culture flasks were incubated at 100 rpm at 28°C, and growth and pigment production was monitored over a period of 72 hours. 5 mL sample was removed every 8 hours, and growth was measured as increase in turbidity at 600 nm. Siderophore was quantified in cell-free supernatant by noting the absorbance at 400 nm using UV-Vis spectrophotometer (Shimadzu UV-2450), and siderophore concentration was calculated as described by Gupta et al. [24].

## 3. Results and Discussion

### 3.1. Viable Counts

**Figure 1:**
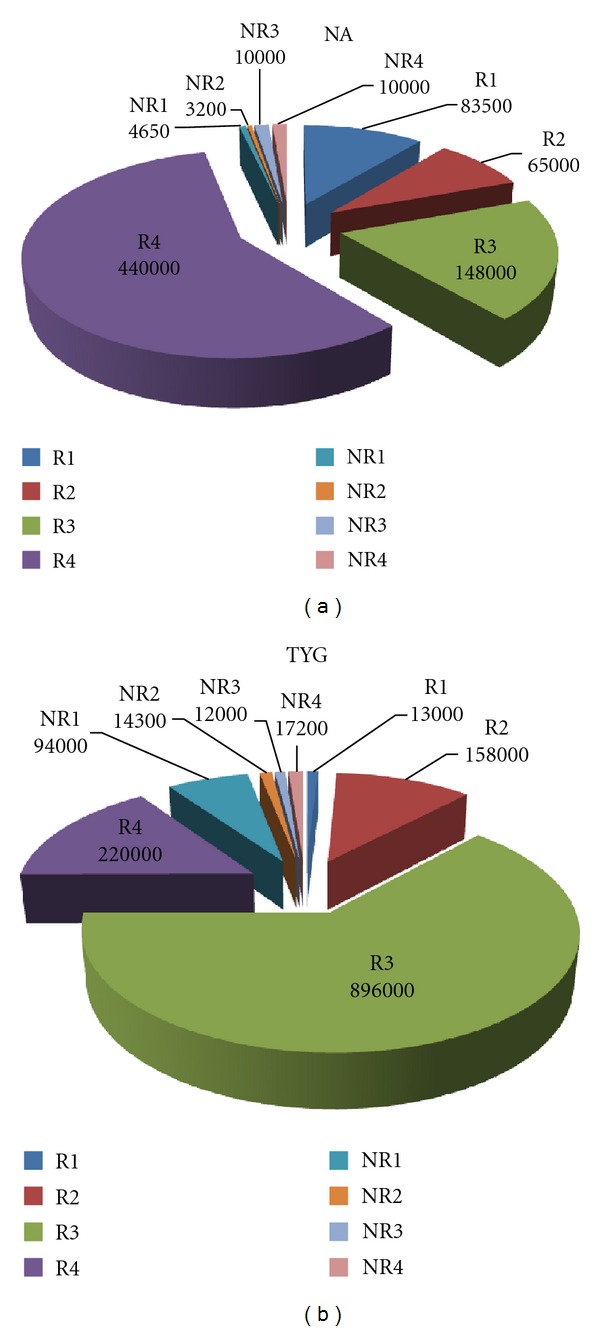
(a) Viable count of the sand dune samples on NA. (b) Viable count of the sand dune samples on TYG.

The total viable count of sand dune samples ranged from 9.4 × 10^3^ to 8.96 × 10^5^ cfu/g on NA (Figure 1(a)). On TYG, viable count ranged from 3.20 × 10^3^ to 8.96 × 10^5^ cfu/g (Figure 1(b)). Higher counts were obtained for rhizosphere samples compared to nonrhizosphere which is expected as plants support microbial growth in their rhizosphere region by increasing release of the exudates [25] or by producing compounds that mimic quorum sensing signals, thus affecting bacterial communities in return [26].

### 3.2. Iron Content of Samples

Iron content of the samples was found to be low and varied from 0.29% to 0.31% (Table 1). Studies on quartz grains from a range of environments from continental and coastal dune fields have reported iron-rich clay coatings on the grains [27]. Sand dunes can have very high Fe content as has been reported in Saharan dust which contains up to 8 percent of Fe_2_O_3_ [28].

### 3.3. Distribution of Siderophore Producers

**Figure 2:**
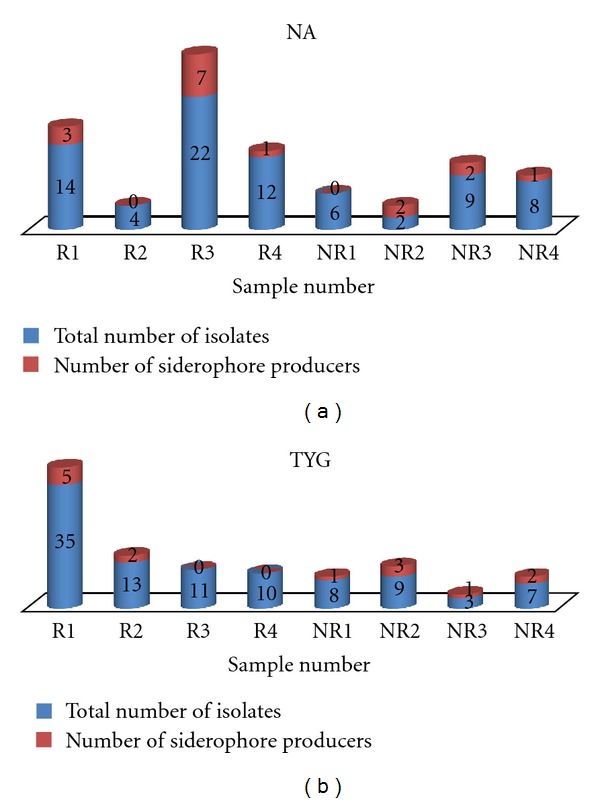
(a) Distribution of the siderophore producers isolated on NA. (b) Distribution of the siderophore producers isolated on TYG.

A total of 173 isolates were obtained, of which 77 were isolated on NA and 96 bacterial strains on TYG. 20.77% of the total NA isolates were siderophore producers, while 14.58% of TYG isolates produced CAS detectable siderophore. Comparative study showed that almost an equal fraction of bacterial population isolated from rhizosphere and nonrhizosphere regions on NA were siderophore producers, while a higher fraction of bacterial population from nonrhizosphere region obtained on TYG were siderophore producers (Figures 2(a) and 2(b)).

### 3.4. Identification of the Selected Isolates

Based on the characteristics, the isolates were identified using Bergey's Manual of Systematic Bacteriology. 16S rRNA gene of nine isolates was sequenced, and the sequences were deposited in GenBank. Of the 30 total siderophore producers isolated, 16 belonged to *Bacillus* spp accounting for 53.3% of the total siderophore producers (Tables 2(a) and 2(b)). 3 isolates each belonged to *Brochothrix*, *Corynebacterium,* and *Renibacterium* species. Two species of *Streptomyces* and *Pseudomonas* and one species of *Kurthia* and *Azotobacter* were detected. The predominance of *Bacillus* spp. could be due to their ability to form siderophores and resist adverse ecological conditions characteristic of sand dune ecosystems [13]. *Bacillus* spp. have been reported to promote plant growth in barley [29], pine and spruce [30], and in eggplant [13]. Shin et al. [9] and Park et al. [5] have also reported predominance of *Pseudomonas* spp. associated with sand dune vegetation.

Our results interestingly showed that the ecosystem harbors a large number of siderophore producers which were detected using the CAS assay. Out of the total 177 isolates, 16.94% showed siderophore production. It was also significant to note that the nonrhizosphere region also harbored siderophore-producing organisms. Anthropogenic material and marine fauna contributes to the organic material which supports growth of microorganisms. However, the survival of such bacteria is again dependent upon their capacity to adapt. The siderophore-producing organisms are just one such group of bacteria which we report from such environment.

### 3.5. Degradation of Sodium Benzoate by the Selected Isolates

**Figure 3:**
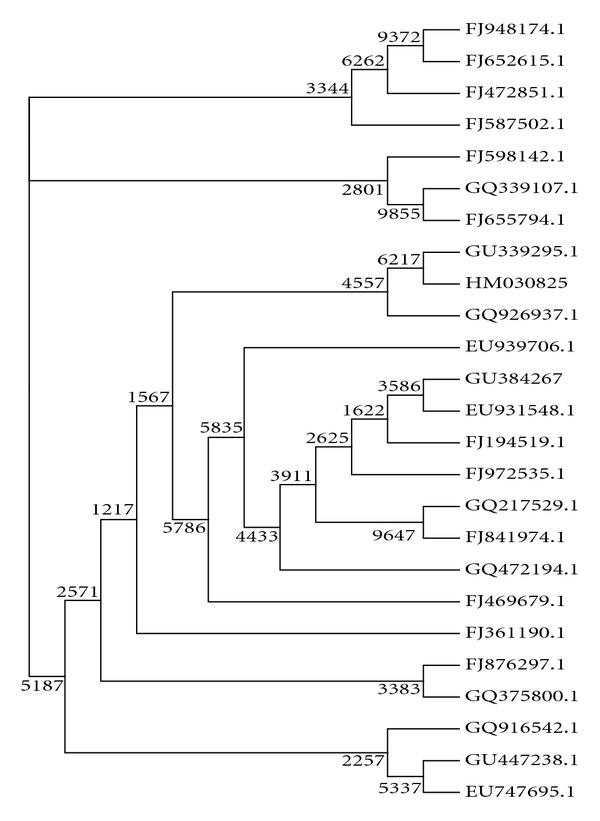
Unrooted tree constructed using the neighbour-joining method showing the phylogenetic relationships of *Pseudomonas aeruginosa* strain TMR2.13 (accession number: HM030825) and other strains of *Pseudomonas aeruginosa*.

Of the 30 siderophore producers, 6 isolates were found to grow using sodium benzoate as the sole source of carbon (Table 3). A significant characteristic of the isolate TMR2.13 was its ability to grow on sodium benzoate as sole source of carbon upto a concentration of 2% along with a production of a yellow green pigment. Phylogenetic analysis of 16S rRNA gene of the isolate (accession number: HM030825) showed 96% similarity (based on BLAST) to *Pseudomonas aeruginosa* with closest similarity to *Pseudomonas aeruginosa *(EH70) (GU339295.1) (Figure 3).

### 3.6. Characteristics of the Siderophore

**Figure 4:**
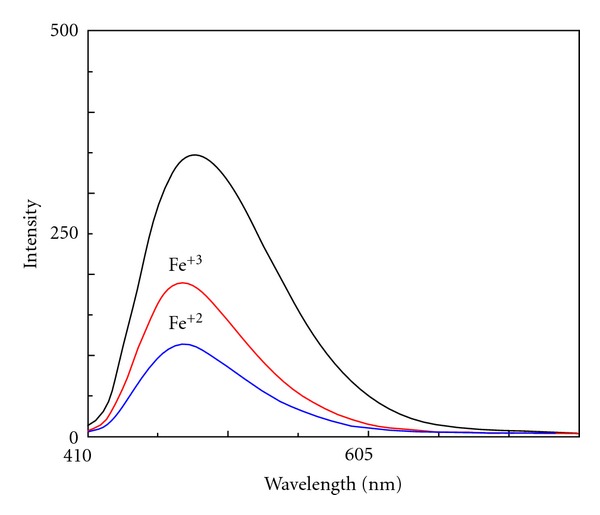
Fluorescence spectra of pyoverdine illustrating fluorescence quenching with Fe^+2^ and Fe^+3^.

Cell-free supernatants of TMR2.13, when scanned in spectrophotometer, showed a sharp peak at 400 nm, and spectrofluorimetric analysis of the supernatants demonstrated excitation and emission wavelengths of 400 and 467 nm, respectively. Further, the fluorescence in the supernatant showed quenching with Fe^+2^ and Fe^+3^ (Figure 4). *Pseudomonas aeruginosa* is reported to produce *pyoverdine,* a yellow fluorescent pigment with strong affinity for iron, and gives absorption maxima at 400 nm and emission at 460 nm [31, 32]. Pyoverdines are the most complex siderophores known to date and represent the primary iron uptake system in fluorescent pseudomonads [33, 34]. Pyoverdine is also reported to have affinity for Fe^+2^ as well as Fe^+3^ with Fe^+2^ binding faster than Fe^+3^ [23]. With the pigment produced by the isolate TMR2.13, Fe^+2^ immediately quenched the fluorescence to 45.58%, while Fe^+3^ quenched the fluorescence to 67.08% within 30 seconds. Xiao and Kisaalita [23] have also demonstrated that pyoverdines also bind and oxidize Fe^+2^ with the binding of pyoverdines to Fe^+2^ being faster than to Fe^+3^ and suggested that the phenomenon could be due to the precipitation of Fe^+3^ as Fe(OH)_3_.

### 3.7. Effect of Sodium Benzoate on Siderophore Production at Different Concentrations of Iron

**Figure 5:**
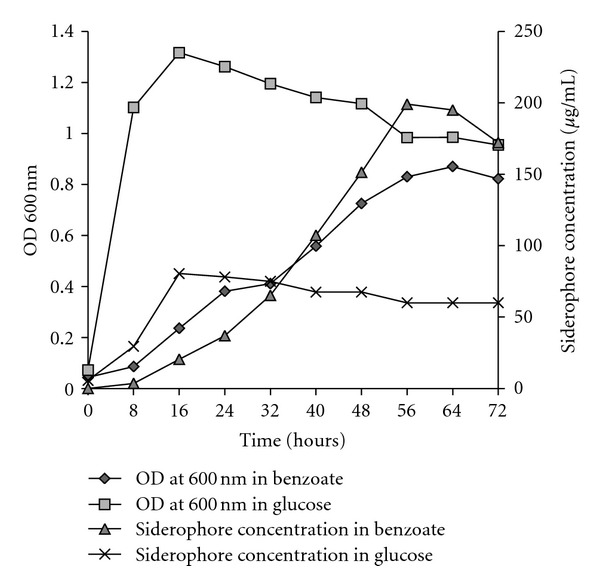
Growth and siderophore production by *Pseudomonas aeruginosa* in MSM with glucose and sodium benzoate without the added Fe.

During the initial stages of growth, the culture produces siderophore, in MSM with glucose or sodium benzoate. Relation between growth and siderophore production showed simultaneous formation of siderophore during exponential growth phase in the medium with no added iron. Further, growth and siderophore production in glucose and benzoate showed significant differences. The production of siderophore was much higher in the presence of benzoate, 199.071 *μ*g/mL, indicating the specific requirement of iron for growth of the organism on the aromatic hydrocarbon (Figure 5).

**Figure 6:**
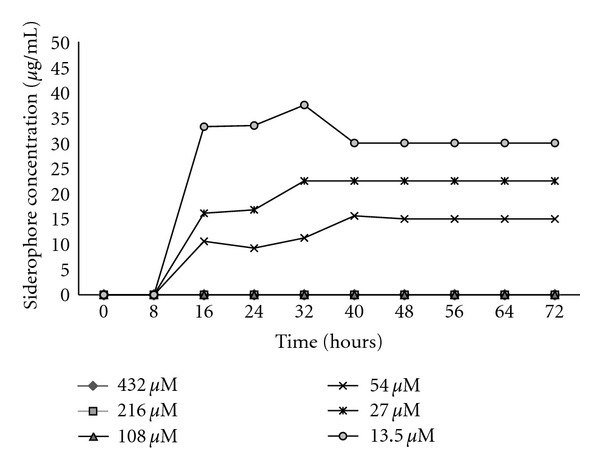
Siderophore production in MSM with glucose as the sole carbon source with different iron concentration (13.5–432 *μ*M).

**Figure 7:**
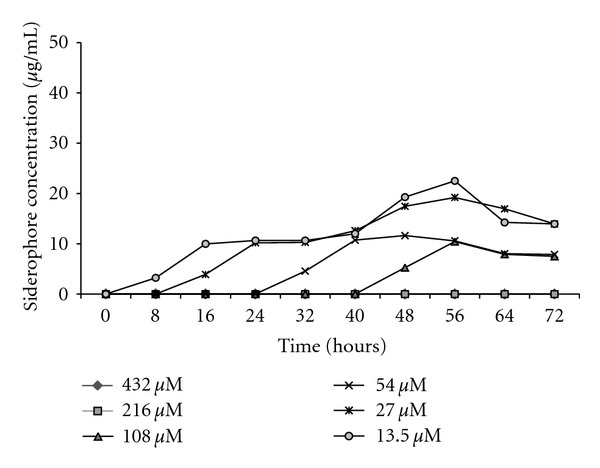
Siderophore production in MSM with sodium benzoate as the sole carbon source with different iron concentration (13.5–432 *μ*M).

In MSM with glucose containing 13, 27, and 54 *μ*M of added iron, the siderophore appeared only after 8 hours (Figure 6). No siderophore was detected in MSM with 108, 216, and 432 *μ*M of added iron indicating that the iron is sufficient to support the growth on glucose. Further, with sodium benzoate as the carbon source, siderophore production was initiated immediately with 13.5 *μ*M of the added Fe^+2^, while in flasks containing 27, 54, and 108 *μ*M of the added iron, the siderophore was detected only after 8, 24, and 40 hours, respectively (Figure 7). Further increase in iron concentration (216 and 432 *μ*M) did not show any siderophore production even up to 72 hrs. This indicated that iron concentrations beyond 54 *μ*M inhibited siderophore production in MSM with glucose. However, the inhibition was noticed at much higher concentration (108 *μ*M) in the presence of benzoate.

A report on the effect of trace element requirement has shown that the iron demand in bacteria increases during the expression of alkane hydroxylase [35].A study on the effect of iron concentration on degradation of toluene by *Pseudomonas* strain reports reduction in the efficiency of the culture when the iron concentration is low [3]. Our observations also suggest the requirement of a higher concentration of iron to sustain growth in benzoate medium. It is a well-established fact that mono- and dioxygenases involved in degradation of aromatic hydrocarbons require iron as the cofactor [3]. Earlier studies on the effect of iron concentration on siderophore production by *P. aeruginosa* have shown siderophore production even with 248 *μ*M of Fe^+3^ [33].

Biosynthesis and secretion of siderophore is known to be related to the requirement of the iron for metabolism of the specific growth substrates. The presence of aromatic compound along with easily metabolisable cosubstrates supports the production of siderophores. Iron concentration is one of the many factors influencing efficient cleanup of aromatic pollutants. In natural environments, the availability of iron is usually low, more so, in marine and coastal ecosystem as well as arid environments where the concentration of iron can be as low as 10^−18 ^M [36]. In such environments, the presence of hydrocarbon will promote higher levels of siderophore production to support the hydrocarbon degradation. A direct implication of increased level of siderophores is on microbial community structure, as these siderophores can also support the growth of non-siderophore-producing bacteria which may be beneficial or pathogenic or nonculturable [37].

## Figures and Tables

**Table 1:** Iron content of sand dune samples.

Sand dune sample	% of Fe
R1	0.3119
R2	0.3098
R3	0.3128
R4	0.3137
NR1	0.3156
NR2	0.3156
NR3	0.2937
NR4	0.2931

**Table:** (a)

Sr. no.	Culture number	Identification
(1)	TMR1.6.1	*Bacillus *spp.
(2)	TMR1.6.2	*Bacillus *spp.
(3)	TMR1.8	*Bacillus *spp.
(4)	TMR1.17	*Bacillus *spp.
(5)	TMR1.18	*Streptomyces *spp.
(6)	NAMR1.6	*Pseudomonas *spp.
(7)	NAMR1.8	*Bacillus *spp.
(8)	TMR2.6	*Streptomyces *spp.
(9)	TMR2.13	*Pseudomonas aeruginosa*
(10)	NAMR3.1	*Bacillus *spp.
(11)	NAMR3.2	*Renibacterium *spp.
(12)	NAMR3.7	*Renibacterium *spp.
(13)	NAMR3.8	*Renibacterium *spp.
(14)	NAMR3.12	*Corynebacterium *
(15)	NAMR3.13	*Corynebacterium*
(16)	NAMR3.15	*Azotobacter *spp.
(17)	NAMR4.8	*Corynebacterium *spp.
(18)	NAMR4.10	*Bacillus *spp.
(19)	TMNR1.4	*Kurthia *spp.
(20)	TMNR2.7.1	*Bacillus *spp.
(21)	TMNR2.7.2	*Bacillus *spp.
(22)	TMNR2.7.3	*Bacillus *spp.
(23)	NAMNR2.1	*Brochothrix *spp.
(24)	NAMNR2.2	*Brochothrix *spp.
(25)	TMNR3.3	*Bacillus *spp.
(26)	NAMNR3.3	*Bacillus *spp.
(27)	NAMNR3.5	*Bacillus *spp.
(28)	TMNR4.1.1	*Bacillus *spp.
(29)	TMNR4.1.2	*Bacillus *spp.
(30)	NAMNR4.4	*Bacillus *spp.

**Table:** (b)

Sr. no.	Culture number	Biochemically identified as	Accession number	Closest match to
(1)	TMR1.6.2	*Bacillus *spp.	JN596242	EU882849.1 *Bacillus subtilis* strain F3–7
(2)	TMR1.17	*Bacillus *spp.	JN596247	HM016080.1 *Bacillus amyloliquefaciens* strain KSU-109
(3)	TMR1.18	*Streptomyces *spp.	JN596248	AB184071.1 *Streptomyces sclerotialus* strain: NBRC 12246
(4)	TMR2.6	*Streptomyces *spp.	JN596249	AB184657.2 *Streptomyces djakartensis* strain: NBRC 15409
(5)	TMR2.13	*Pseudomonas aeruginosa*	HM030825	GU339295.1 *Pseudomonas aeruginosa* strain EH70
(6)	NAMR4.10	*Bacillus *spp.	JN596242	EU746420.1 *Bacillus* sp. A-34
(7)	TMNR2.7.1	*Bacillus *spp.	JN596243	HQ238533.1 *Bacillus tequilensis* strain S12Ba-171
(8)	TMNR2.7.2	*Bacillus *spp.	JN596244	HQ238638.1 *Bacillus tequilensis* strain S433Ba-70
(9)	TMNR3.3	*Bacillus *spp.	JN596245	GU181234.1 *Bacillus amyloliquefaciens* strain SRDM2

**Table 3:** Growth of isolates on MSM with sodium benzoate.

Sr. no.	Culture number	Growth on sodium benzoate	Maximum sodium benzoate concentration at which the isolates exhibit growth
(1)	TMR1.6.1	−	
(2)	TMR1.6.2	−	
(3)	TMR1.8	−	
(4)	TMR1.17	−	
(5)	TMR1.18	−	
(6)	NAMR1.6	−	
(7)	NAMR1.8	−	
(8)	TMR2.6	−	
(9)	TMR2.13	+	(2%)
(10)	NAMR3.1	−	
(11)	NAMR3.2	−	
(12)	NAMR3.7	−	
(13)	NAMR3.8	−	
(14)	NAMR3.12	−	
(15)	NAMR3.13	−	
(16)	NAMR3.15	−	
(17)	NAMR4.8	−	
(18)	NAMR4.10	−	
(19)	TMNR1.4	−	
(20)	TMNR2.7.1	+	(0.1%)
(21)	TMNR2.7.2	−	
(22)	TMNR2.7.3	−	
(23)	NAMNR2.1	−	
(24)	NAMNR2.2	−	
(25)	TMNR3.3	−	
(26)	NAMNR3.3	+	(0.1%)
(27)	NAMNR3.5	+	(1%)
(28)	TMNR4.1.1	+	(1%)
(29)	TMNR4.1.2	−	
(30)	NAMNR4.4	+	(1%)

Key: +: growth, −: no growth.
